# The orthosteric agonist 2-chloro-5-hydroxyphenylglycine activates mGluR5 and mGluR1 with similar efficacy and potency

**DOI:** 10.1186/1471-2210-12-6

**Published:** 2012-05-29

**Authors:** Paul J Kammermeier

**Affiliations:** 1 Department of Pharmacology and Physiology, University of Rochester Medical Center, Rochester, NY, 14642, USA

## Abstract

**Background:**

The efficacy, potency, and selectivity of the compound 2-Chloro-5-hydroxyphenylglycine (CHPG), a nominally selective agonist for metabotropic glutamate receptor 5 (mGluR5), were examined with select mGluRs by examining their ability to induce modulation of the native voltage dependent ion channels in isolated sympathetic neurons from the rat superior cervical ganglion (SCG). SCG neurons offer a null mGluR-background in which specific mGluR subtypes can be made to express via intranuclear cDNA injection.

**Results:**

Consistent with previous reports, CHPG strongly activated mGluR5b expressed in SCG neurons with an apparent EC_50_ around 60 μM. Surprisingly, CHPG also activated two mGluR1 splice variants with a similar potency as at mGluR5 when calcium current inhibition was used as an assay for receptor function. No effect of 1 mM CHPG was seen in cells expressing mGluR2 or mGluR4, suggesting that CHPG only activates group I mGluRs (mGluR1 and 5). CHPG was also able to induce modulation of M-type potassium current through mGluR1, but not as consistently as glutamate. Since this channel is modulated through a G_q_-dependent pathway, these data indicate that CHPG may exhibit some biased agonist properties on mGluR1. Closer examination of the voltage-independent, Gq-mediated component of mGluR-induced calcium current modulation data confirmed that some biased agonism was evident, but the effect was weak and inconsistent.

**Conclusions:**

These data contrast with the established literature which suggests that CHPG is a selective mGluR5 agonist. Instead, CHPG appears to act equally well as an agonist at mGluR1. While some weak biased agonism was observed with CHPG acting on mGluR1, but not mGluR5, favoring G_i/o_ signaling over G_q/11_, this effect does not appear sufficient to fully explain the discrepancies in the literature.

## Background

Metabotropic glutamate receptors (mGluRs) are important mediators of learning and excitability [1,2], sensory signal transduction [3], and central information processing that play a critical role in many physiological and pathological processes [4]. The group I mGluRs, which includes mGluR1 and 5, exhibit widespread expression in the central nervous system where they are generally expressed postsynaptically in addition to their dendro-somatic localization, where they can initiate some forms of plasticity and regulate neuronal excitability, respectively [1,2]. mGluR1 and 5 are similar in sequence, G protein coupling and in their responses to many pharmacological compounds. While their expression profiles in the brain are distinct, mGluR1 and 5 serve analogous roles in different regions. For example, hippocampal mGluR5 localized near the postsynaptic density can initiate a form of long term depression (mGluR-LTD) [1,5,6]. Likewise, postsynaptic mGluR1 in the cerebellum can also produce mGluR-LTD [1,2,7]. In both cases, initiation of plasticity requires coupling to G_q/11_ proteins and post-synaptic localization, although the mechanistic details of each phenomena are distinct. Further, both mGluR1 and 5 can couple to modulation of voltage gated calcium and other ion channels in several neuronal cell types [8-11], leading to changes in cell excitability.

Our understanding of the role of these receptors derives in large part from the pharmacological tools used to manipulate their function. In recent years, many highly selective compounds have been developed that target mGluRs at allosteric sites, separate from the endogenous glutamate ligand binding site. Fewer highly selective orthosteric compounds are available. One exception is the compound 2-Chloro-5-hydroxyphenylglycine (CHPG), an orthosteric ligand that has been used extensively as a selective mGluR5 agonist [12]. At least one report indicates that CHPG is selective for mGluR5 over even the closely related mGluR1, making it the only known orthosteric agonist with such selectivity [12]. However, the selectivity of CHPG for mGluR5 over mGluR1 has not been subsequently tested, to my knowledge.

Here, selectivity of CHPG was examined by testing its ability to activate specific mGluRs expressed by intranuclear cDNA injection in sympathetic neurons from the rat superior cervical ganglion (SCG), a primary neuronal cell with a null mGluR background [13]. Modulation of the native voltage-dependent calcium currents in SCG neurons was used as an assay for heterologously expressed mGluRs. The ability of a range of concentrations of CHPG to activate mGluR5, mGluR2, mGluR4, and two splice variants of mGluR1 (a and b) was tested. Consistent with previous reports in the literature, we found that CHPG functioned as a full agonist at mGluR5 and failed to activate mGluR2, or mGluR4. Surprisingly however, CHPG also functioned as a full agonist at both mGluR1a and mGluR1b with similar potency as mGluR5.

## Methods

### Cell isolation, DNA injection and Plasmids

A description of cell isolation and cDNA injection is found elsewhere [14]. Animal protocols were approved by the university committee on animal resources (UCAR). Briefly, SCGs were removed from adult male Wistar rats (175–225 g) after CO_2_ euthanasia and decapitation, then incubated in Earle’s balanced salt solution (InVitrogen, Life Technologies Carlsbad, CA) containing 0.6 mg/ml trypsin (Worthington Biochemicals, Freehold, NJ) & 0.8 mg/ml collagenase D (Boehringer Mannheim Biochemicals, Indianapolis, IN) for 60 min at 35°C. Cells were transferred to minimum essential medium (InVitrogen/Gibco), plated on poly-l-lysine (Sigma Chemical Co., St. Louis, MO) coated culture dishes and incubated at 37°C for 2–4 hours before cDNA injection. Injected cells were incubated overnight at 37°C (95% air and 5% CO_2_; 100% humidity) and patch clamp experiments were performed the next day.

Injection of cDNA was performed with an Eppendorf 5247 microinjector and InjectMan NI 2 micromanipulator (Madison, WI) 3–5 hours following cell isolation. Injection electrodes were made with a Sutter P-97 horizontal electrode puller (Novato, CA) from thin-walled, borosilicate glass (World Precision Instruments, Sarasota, FL). Plasmids were stored at −20°C as a 0.4 - 1 μg/μl stock solution in TE buffer (10 mM TRIS, 1 mM EDTA, pH 8). All mGluR constructs were injected at 100–130 ng μl^-1^ (pCDNA3.1^+^; InVitrogen). The mGluR4 clone was provided by D. Hampson (University of Toronto, Toronto, Onatrio, Canada). All neurons were co-injected with “enhanced” green fluorescent protein cDNA (0.02 μg/μl; pEGFPN1; BD Biosciences-Clontech, Palo Alto, CA) for identification of successfully injected cells.

All constructs were sequence confirmed. PCR products were purified with Qiagen (Valencia, CA) silica membrane spin columns prior to restriction digestion and ligation. Midipreps were prepared using Qiagen anion exchange columns, and amplified in either Top10 or DH5α E. coli. (InVitrogen).

### Electrophysiology and data analysis

Pipettes for patch-clamp experiments were made with a Sutter P-97 horizontal puller from 8250 glass (Garner Glass, Claremont, CA) and had resistances of 1–3 MΩ. Series resistances were 2.3 ± 0.2 MΩ (n = 31) prior to electronic compensation of 80%. Whole-cell patch-clamp recordings were made with an EPC-7 patch clamp amplifier (Heka Elektronik, Germany). Voltage protocol generation and data acquisition were performed using custom software (courtesy Stephen R. Ikeda, NIAAA, Rockville, MD) on a Macintosh G4 computer (Apple Computer, Cupertino, CA) with an InstruTech (Port Washington, NY; now Heka Elektronik) ITC-16 data acquisition board. Currents were low-pass filtered at 3 kHz using the 4-pole Bessel filter in the patch clamp amplifiers, digitized at 2–5 kHz and stored on the computer for later analysis. Experiments were performed at 21–24°C (room temperature). Patch-clamp data analysis was performed using the Igor Pro software package (Wavemetrics, Lake Oswego, OR).

The external (bath) recording solution contained (in mM): 155 tris hydroxymethyl aminomethane, 20 4-(2-Hydroxyethyl)-1-piperazineethanesulfonic acid (HEPES), 10 glucose, 10 CaCl_2_, and 0.0003 tetrodotoxin (TTX), pH 7.4. The internal (pipette) solution contained: 120 N-methyl-D-glucamine (NMG) methanesulfonate, 20 TEA, 11 EGTA, 10 HEPES, 10 sucrose, 1 CaCl_2_, 4 MgATP, 0.3 Na_2_GTP, and 14 tris creatine phosphate, pH 7.2. l-Glutamate (Sigma) was used as the agonist for mGluRs. CHPG was obtained from two sources: Tocris Bioscience (Ellisville, MO) and Ascent Scientific (Avonmouth, Bristol, UK). All drugs and control solutions were applied to cells using a custom, gravity-driven perfusion system positioned ~100 μm from the cell, allowing rapid solution exchange (≤ 250 ms). The degree of calcium current inhibition was calculated as the maximum current inhibition in the presence of drug compared to the last current measurement prior to drug application.

## Results

### CHPG activates mGluR5

**Figure 1:**
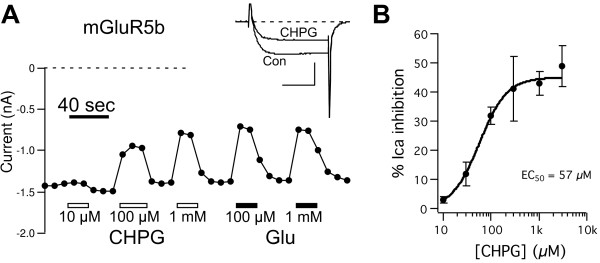
**CHPG induces strong calcium current modulation in SCG neurons expressing mGluR5.A**, Time course and sample current traces (*inset*) illustrating the magnitude of calcium currents in a representative SCG neuron and inhibition by various concentrations of CHPG and Glu, as indicated. Voltage protocol consisted of a 25 msec step to +10 mV from a holding potential of −80 mV. Scale bars in inset represent 1 nA and 10 msec. **B**, Average (±SEM) calcium current inhibition at a range of CHPG concentrations (for [CHPG] ranging from 10 μM to 3 mM, n = 6, 3, 8, 4, 11, and 7, respectively).

To confirm the utility of CHPG as an agonist of mGluR5, and to estimate its efficacy and potency in our system, isolated SCG neurons were intranuclearly injected with cDNA encoding rat mGluR5b, and the amplitude of calcium currents was monitored using the whole-cell configuration of the patch-clamp technique upon application of multiple concentrations of CHPG and L-glutamate (Glu) (Figure 1). Currents were monitored during a 25 msec test pulse to +10 mV from a holding potential of −80 mV (Figure 1A, inset), near the peak of the current–voltage (I-V) curve. CHPG application produced a dose-dependent, reversible inhibition of the calcium current, as illustrated in the representative current amplitude time course shown in Figure 1A. At saturating concentrations the CHPG effect was similar to the inhibition produced with Glu (Figure 1A, B). Figure 1B shows the average (±SEM) current inhibition by CHPG at each concentration. A fit of these data to the Hill equation {% inhibition = E_MAX_/(1+ [[CHPG]_half_/[CHPG]^rate^)} is also shown, yielding an EC_50_ value of 57 μM. These data are consistent with those reported in the literature and confirm that CHPG acts as an agonist at mGluR5 with an EC_50_ value in the mid to high μM range.

### Effect of CHPG on other mGluRs

**Figure 2:**
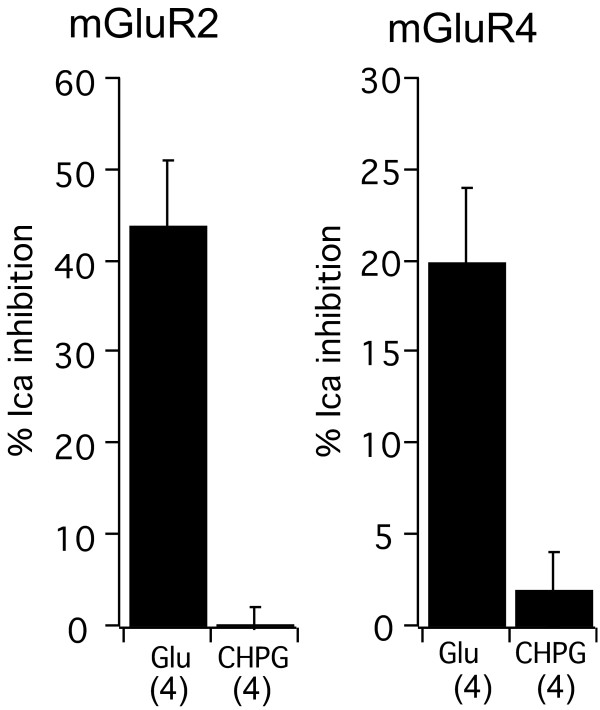
**CHPG does not activate group II or III mGluRs.** Average (± SEM) calcium current inhibition by 100 μM Glu and 1 mM CHPG in SCG neurons expressing mGluR2 (*left*) or mGluR4 (*right*). Both Glu and CHPG were applied to each cell. Number of cells in each group shown in parentheses.

The specificity of CHPG was tested by examining its effect on calcium currents in neurons expressing either mGluR2, mGluR4, mGluR1a or mGluR1b. SCG neurons expressing either mGluR2 or mGluR4, group II and III mGluRs, respectively, which couple exclusively to G_i/o_ proteins and with greater sequence divergence from mGluR5 (compared to mGluR1), were exposed to 100 μM Glu and 1 mM CHPG. All of the mGluR2-expressing cells examined showed strong Glu-dependent calcium current inhibition (average of 44 ± 7%, n = 4), confirming expression of the receptor (Figure 2). However, none of these cells exhibited detectable current modulation upon application of 1 mM CHPG (0 ± 2%, n = 4). Likewise, SCG neurons expressing mGluR4 were inhibited 20 ± 4% (n = 4) by 100 μM Glu, but unaffected by 1 mM CHPG (2 ± 2%, n = 4). These data confirm that CHPG is not an agonist at mGluR2 or 4.

**Figure 3:**
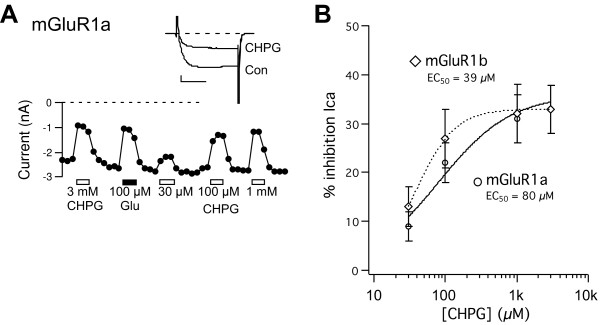
**CHPG induces strong calcium current modulation in SCG neurons expressing mGluR1a and 1b.A**, Time course and sample current traces (*inset*) illustrating the magnitude of calcium currents in a representative mGluR1a expressing SCG neuron and inhibition by various concentrations of CHPG and by 100 μM Glu, as indicated. Cells were held at −80 mV and current was elicited by a 25 msec step to +10 mV. Scale bars represent 0.5 nA and 10 msec. **B**, Dose response curves for CHPG in cells expressing mGluR1a (open circles) or mGluR1b (open diamonds, as indicated. For mGluR1a, n = 12 at all concentrations. For mGluR1b (30–3000 μM), n = 5, 6, 7 and 7, respectively.

The effects of CHPG were further tested on SCG neurons expressing either mGluR1a or a similar splice variant, mGluR1b. Surprisingly, strong CHPG-mediated calcium current modulation was observed in neurons expressing both mGluR1a and mGluR1b (Figure 3A, B). Figure 3A illustrates the time course of inhibition in one SCG neuron expressing mGluR1a upon application of 100 μM Glu and several concentrations of CHPG (as indicated). The inset in Figure 3A shows sample currents from the same cell illustrating a control (uninhibited) current, and current inhibited by 3 mM CHPG. Figure 3B illustrates the average (± SEM) CHPG effect at four different concentrations from 12 SCG neurons expressing mGluR1a and 7 cells expressing mGluR1b. The data were fit to the Hill equation (as in Figure 1B), which indicated an EC_50_ value of 80 μM for mGluR1a and 39 μM for mGluR1b, both values similar to that observed with mGluR5 (Figure 1). These data suggest that while CHPG shows strong selectivity for group I mGluRs over mGluR2 and 4, it appears to activate mGluR1 with similar potency as its known target, mGluR5.

### Efficacy of CHPG on mGluR5 and mGluR1

**Figure 4:**
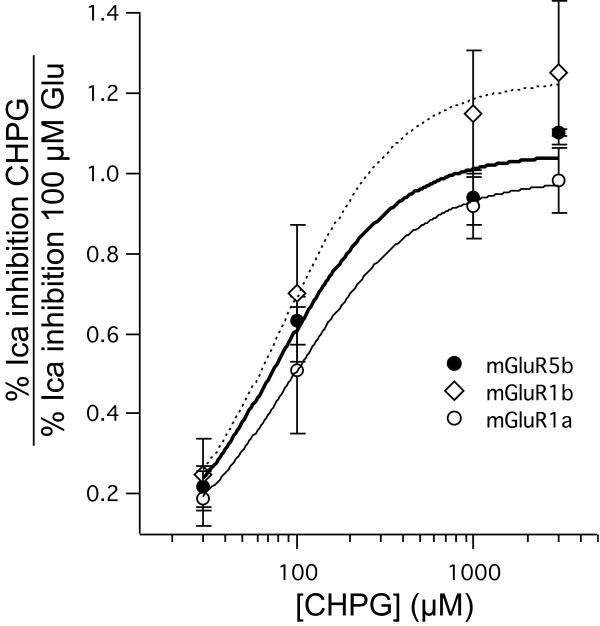
**Efficacy and potency of CHPG were similar for SCG calcium current modulation in cells expressing mGluR5b, mGluR1a, and mGluR1b.** Calcium current inhibition by each concentration of CHPG was normalized to that by 100 μM Glu in each cell, and plotted here. Neither efficacy nor potency of CHPG’s effects on mGluR1a, 1b or 5b were statistically distinguishable.

To compare the efficacy of CHPG on mGluR5 with the mGluR1 variants, the responses (percentage inhibition of the calcium current) to each concentration of CHPG were normalized to the response to 100 μM Glu in the same cell. These normalized CHPG responses were then averaged and plotted in Figure 4. Because 100 μM Glu is a saturating concentration of the natural ligand (and full agonist) of mGluRs, a saturating response of 1 in this plot is indicative of full agonism. Each of these data sets were fit to the Hill equation and responses to mGluR5, mGluR1a, and mGluR1b reached a maximal response of 1.05 ± 0.09 (n = 8), 0.99 ± 0.02 (n = 12), and 1.23 ± 0.04 (n = 7), respectively. These data suggest that CHPG acts as a full agonist at mGluR5, mGluR1a and mGluR1b. Note that the number of cells in the mGluR5 data set was somewhat smaller than in Figure 1, because only cells to which 100 μM Glu was applied could be analyzed in this way.

**Figure 5:**
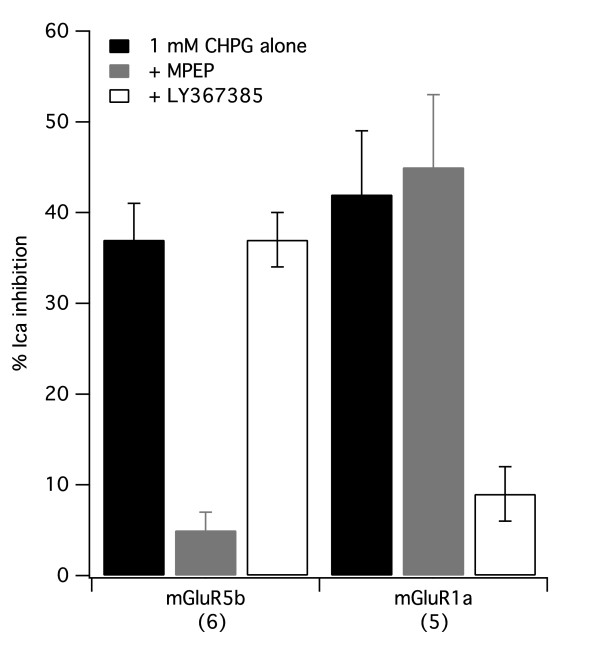
**Effect of selective inhibitors on CHPG effects in mGluR5b and 1a expressing cells.** Average (± SEM) calcium current inhibition produced by application of 1 mM CHPG alone (*black*) or in the presence of 1 μM MPEP (*gray*) or 50 μM LY367385 (*white*) in SCG neurons expressing the indicated receptor. Number of cells in each group shown in parentheses.

To further verify the action of CHPG on these receptors, calcium current inhibitory responses of SCG neurons to 1 mM CHPG were examined in cells expressing either mGluR5b or 1a in the absence and presence of the selective mGluR5 inhibitor MPEP and the selective mGluR1 inhibitor LY367385. The results of those experiments are shown in Figure 5. In six mGluR5b expressing cells, 1 mM CHPG produced an average inhibition of the calcium currents of 37 ± 4% when applied alone. In the presence of 1 μM MPEP, CHPG produced only 5 ± 2% inhibition, as expected. When CHPG was applied in the presence of 50 μM LY367385, CHPG inhibited the current by 37 ± 3%, an effect indistinguishable from that when CHPG was applied alone. In five mGluR1a expressing cells, CHPG produced a 45 ± 7% inhibition when applied alone, and 45 ± 8% and 9 ± 3% in the presence of MPEP and LY367385, respectively. These results confirm that CHPG is indeed acting through the heterologously expressed receptor in both mGluR5 and mGluR1 expressing SCG neurons, and are thus inconsistent with the plausible explanation that native mGluR5 might be upregulated in SCG neurons when mGluR1 is expressed heterologously.

### CHPG shows weak biased agonist properties when acting on mGluR1 but not mGluR5

**Figure 6:**
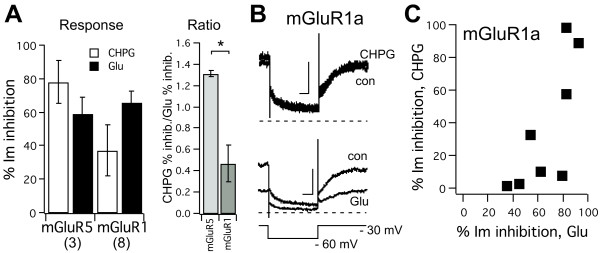
**Modulation of M-current by CHPG in SCG neurons expressing either mGluR5b or mGluR1a.A**, *Left*, Average (±SEM) calcium current by 1 mM CHPG (*open*) and 100 μM Glu (*filled*), in SCG neurons expressing mGluR5b or mGluR1a, as indicated. Number of cells in each group is shown in parentheses. *Right*, Average (±SEM) ratio of CHPG/Glu inhibition of calcium current in the same cells as in *Left*. Asterisk indicates p < 0.05, *T*-test. Note that since ratios are non-linearly distributed around unity, statistical tests were performed on the natural log of the ratio (CHPG/Glu inhibition) from each individual cell. **B**, Sample current traces illustrating M-current inhibition in an mGluR1a expressing cell. In this cell, CHPG had little effect while Glu strongly inhibited the current. M-currents were measured as the tail current deactivating during a hyperpolarizing step to −60 mV from a holding potential of −30 mV (to inactivate other K^+^ channels). Voltage protocol is shown below. **C**, Distribution of percent inhibition by CHPG plotted against the inhibition by Glu in each cell.

A potential explanation for the discrepancy between the data presented here, showing CHPG acting as an mGluR1 agonist, and that in the established literature suggesting that CHPG is selective for mGluR5 over mGluR1, may be that CHPG acts as a biased agonist on mGluR1. This is possible because although the group I mGluRs (mGluR1/5) are generally considered to be G_q_-coupled receptors, they are in fact dual coupled, activating G_q/11_ and G_i/o_ in many neurons [8-11], or G_q/11_ and G_s_ in some heterologous expression systems [15-17]. As such it is possible that mGluR1 agonism was missed in earlier studies, since many of these used fluorescent calcium indicators to detect mGluR-induced rises in intracellular calcium as an assay for mGluR5 and mGluR1 function, which depends exclusively on G_q/11_ activation. If CHPG functions as a biased agonist on mGluR1, producing activation of only the G_i/o_ pathway, this activity would have been missed. To assess whether CHPG could activate G_q/11_ as effectively as Glu in cells expressing mGluR5b or mGluR1a, modulation of the native M-type potassium currents in SCG neurons was examined. M-currents are non-inactivating, voltage-dependent potassium currents that are strongly inhibited by G_q/11_ activation [18-20]. In SCG neurons, KV7.2 and KV7.3 (KCNQ2 and KCNQ3) subunits underlie the M-current [21], and their inhibition is due to depletion of PIP_2_ levels by phospholipase C [19,20]. Thus, M-current inhibition by mGluR1 and 5 functions as a pure assay for G_q/11_ activation, as there is no pertussis toxin sensitive (G_i/o_) component to this modulation [11]. As expected, SCG neurons expressing mGluR5b were strongly inhibited by both 1 mM CHPG and 100 μM Glu (Figure 6A). In these cells, the M-current was inhibited by 78 ± 13%, and 59 ±10% by CHPG and Glu, respectively (n = 3). In 8 mGluR1a-expressing cells examined, modulation of the M-current by Glu was strong, as expected, with an average inhibition of 67 ± 8%. However, the inhibition produced by CHPG in the same cells was quite variable and on average, much smaller than that produced by Glu. CHPG inhibition via mGluR1a averaged only 37 ± 15% (Figure 6A, B). Indeed, every cell examined showed substantial Glu-mediated M-current inhibition, but only half of these cells exhibited detectable inhibition by CHPG. This is evident when CHPG inhibition was plotted vs. Glu inhibition for each cell in Figure 6C. Indeed, the ratio of CHPG-mediated inhibition to Glu mediated inhibition of the M-current was significantly smaller in mGluR1a expressing SCG neurons than in mGluR5b expressing cells (Figure 6A *right*), suggesting that CHPG does show some degree of partial agonism in this system. These data may help to explain the discrepancy between the data presented here, suggesting that CHPG functions as an agonist with similar efficacy and potency on mGluR1a as on mGluR5, and that in the literature, which reports highly selective effects of CHPG [12]. It should be emphasized however, that CHPG was not a poor activator of this pathway in every mGluR1a expressing cell, suggesting that the apparent biased agonism is complex and may be influenced by other, as yet undetermined factors.

**Figure 7:**
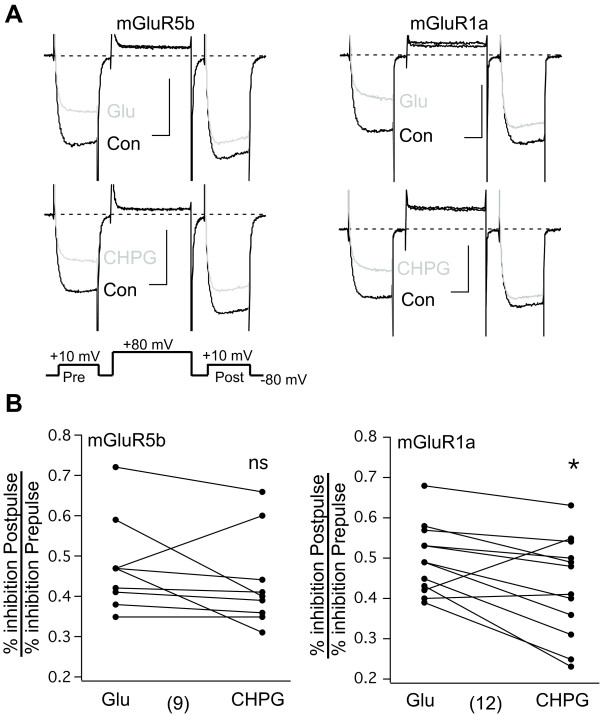
**DIfferential mechanism of calcium current modulation by Glu and CHPG in cells expressing mGluR1a.A**, Sample calcium current traces elicited with the voltage protocol shown (*below*) illustrating control and Glu (*upper*) or CHPG (*lower*) inhibited currents in cells expressing either mGluR5b (*left*) or mGluR1a (*right*). Inhibited traces shown in gray. Note that the G_q_-mediated component of inhibition is not reversed by the depolarizing step, and is therefore similar in both “Pre” and “Post.” Scale bars represent 0.4 nA and 10 msec. **B**, Summary of the G_q_-mediated, voltage independent component of inhibition (expressed as the Post/Pre inhibition ratio) for Glu and CHPG (paired measurements in each cell) from mGluR5 (left) and mGluR1 (right) expressing cells. Number of cells in each group shown in parentheses.

To further explore the potential biased agonist properties of CHPG on mGluR1, the calcium current inhibition via CHPG was examined in more detail. Modulation of SCG calcium currents by group I mGluRs is complex. Activation of both G_i/o_ and G_q/11_ by mGluR1 and 5 leads to calcium current inhibition, and the two pathways are distinguishable by their voltage dependence. The pathway initiated by G_i/o_ activation is mediated by Gβγ [22], and is therefore at least partially reversible following a strong depolarizing step [23]. By contrast, the G_q/11_ mediated inhibition is voltage independent [11]. To evaluate the relative contributions of these modulatory pathways, a triple pulse voltage protocol can be used to monitor current amplitude as the receptors are activated [24] (Figure 7A). This protocol consists of two test pulses to +10 mV separated by a 50 msec depolarizing step to +80 mV. Because the depolarizing step will selectively reverse much of the G_i/o_-mediated inhibition due to its voltage dependence, the relative contribution of each G protein pathway to calcium current inhibition can be gauged by simply dividing the percent inhibition in the second test pulse (“Post” in Figure 7A) by that in the first (“Pre”). The higher this inhibition ratio, the stronger the relative contribution of the G_q/11_ pathway. However, because the voltage dependent pathway is not completely reversed by the depolarizing step and because the relationship between the two pathways is not strictly additive [25], this method cannot be used to determine the absolute contribution of each pathway. Nevertheless, to determine whether CHPG was a weaker activator of G_q/11_ then Glu when acting through mGluR1a, the Post/Pre inhibition ratio was examined during calcium current inhibition experiments in SCG neurons expressing either mGluR5b or mGluR1a (Figure 7).

As noted previously [11], the contribution of the voltage dependent and voltage independent calcium current inhibitory pathways via group I mGluRs in SCG neurons is quite variable. Further, based on the M-current data (Figure 6), it was expected that any differences in G_q/11_ activation by CHPG and Glu would be inconsistent. Therefore, to insure that any changes would be detectable, analysis was restricted to cells in which the Post/Pre inhibition ratio by 100 μM Glu was 0.35 or higher, because these cells had a clearly detectable G_q/11_ component. Figure 7A shows sample current traces illustrating the voltage dependence of inhibition by 100 μM Glu (upper) and 1 mM CHPG (lower) in SCG neurons expressing either mGluR5b (left) or mGluR1a (right). Figure 7B shows a plot of the Post/Pre inhibition ratio for Glu and CHPG in each group for every cell in which the ratio was > 0.35. While there was substantial variability in the ratios from cell to cell, and in the differences in ratios between Glu and CHPG, the Post/Pre inhibition ratios for Glu and CHPG in mGluR5-expressing cells was statistically indistinguishable. A small but statistically significant difference was observed however, in mGluR1a expressing cells (paired *T*-test, p ≤ 0.05) when comparing the inhibition by CHPG to that by Glu by providing more evidence that CHPG is a poorer activator than Glu of the G_q/11_ pathway in some cells. These data lend some support for the ability of CHPG to act as a biased agonist on mGluR1, but both the calcium current and M-current inhibition data demonstrate that CHPG is capable of activating G_q/11_, even fairly strongly, in some cells.

## Discussion

The data presented here indicate that contrary to previous reports [12], the nominally selective agonist CHPG can activate mGluR1 with similar efficacy and potency as mGluR5. Consistent with the literature however, CHPG did not produce any detectable activation of mGluR2 or mGluR4. The effects of CHPG were examined using heterologous expression of each receptor in rat sympathetic neurons, an adult neuronal cell type with null-mGluR expression, and assayed using G protein mediated modulation of native ion channel currents as an assay for receptor signaling. Further, using M-current inhibition as an assay for receptor function, a pathway that depends only on G_q_ signaling, revealed that in some cells, CHPG agonism of mGluR1 appeared to show some biased agonism. Specifically, while some mGluR1-expressing cells showed similar M-current inhibition using CHPG or Glu as an agonist, others were strongly inhibited by Glu, but only very weakly by CHPG. These data provide some contrast to those obtained using calcium current inhibition as an assay, which proceeds through a combination of G_q/11_ and pertussis toxin sensitive Gβγ activation [11]. Examination of calcium current inhibition by CHPG in mGluR1 expressing cells revealed that the G_q/11_-mediated, voltage independent inhibitory pathway was not as strongly activated by CHPG as Glu, supporting the hypothesis that CHPG is a poorer G_q/11_ activator than Glu. However, the difference was not robust. Thus, while these data indicate that CHPG can effectively act as an agonist at mGluR1a and its splice variant mGluR1b, the overall balance of G_i/o_/G_q/11_ protein activation may be altered when CHPG rather than Glu is used as the agonist.

**Figure 8:**
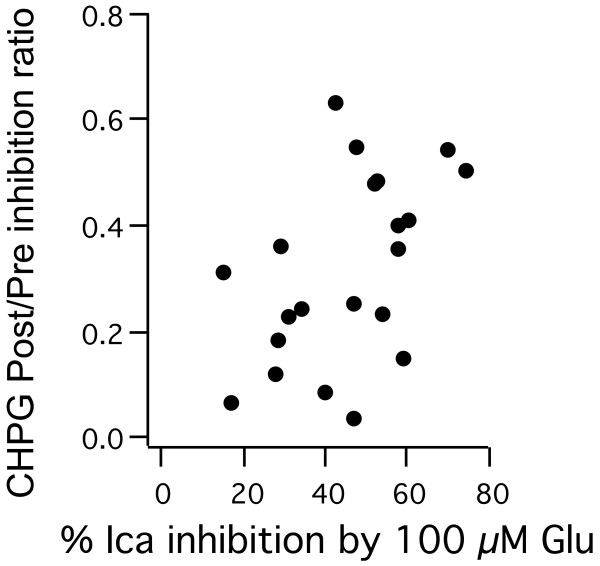
**Relative strength of G**_**q**_**activation by CHPG is not well correlated with apparent mGluR1 expression level.** Relative strength of the G_q_ inhibitory pathway, expressed as the Post/Pre inhibition ratio vs. apparent mGluR1 expression level, expressed as magnitude of inhibition by 100 μM Glu in each cell. Note that all of the mGluR1 expressing cells recorded are shown, not just those with strong G_q_ components, as in Figure 7.

The apparent biased agonism of CHPG when applied to mGluR1-expressing cells was interesting, but the variability of the effect is difficult to explain. It is possible that the variability was related to mGluR1 expression level, but this conclusion is difficult to reconcile with the data. Due to the nature of these studies, receptor expression cannot be directly measured, but an estimate of expression can be made by assuming that expression level correlates with the magnitude of inhibition by Glu in each cell. For example, the M-current inhibition data in Figure 6 shows that in the 8 mGluR1a expressing cells examined, M-current inhibition ranged from about 35% to about 90%. While there may be some relationship between CHPG responses and Glu responses in these cells, the relationship is weak. For example, in the 4 cells that showed virtually no response to CHPG, the range of Glu responses was quite broad, including two cells with substantial inhibition by Glu (~ 60 and 80%). Further, examination of the voltage dependence of calcium current inhibition (Figure 7) yields similar, ambiguous results (Figure 8). If the Post/Pre calcium current inhibition ratio, a measure of the strength of Gq activation by CHPG, is plotted against the amplitude of total inhibition by Glu, an estimate of expression level, the effects are widely scattered and poorly correlated (Figure 8). Thus, the data do not provide strong evidence that the degree of G protein activation bias by CHPG via mGluR1 is related to receptor expression levels. It should also be noted that while CHPG was less efficient than Glu at producing G_q_-mediated current inhibition in most of the mGluR1 expressing cells examined in the calcium current studies, CHPG still activated this pathway to a relatively strong degree (Figures 7, 8). Therefore, while some bias in G protein activation of CHPG via mGluR1 is detectable, it is unlikely that this effect will be meaningful in a physiological or in vivo context. However, the existence of a group I mGluR agonist with G protein bias is novel, and suggests that development of compounds with more significant biased properties is possible.

Given the effect of CHPG on mGluR1a, it was not surprising that the drug had similar effects on mGluR1b since both splice variants are identical in the N-terminal, ligand binding region. In fact, these proteins differ only in their extreme cytoplasmic C-termini, which is not expected to alter receptor pharmacology. Furthermore, there is no evidence that G protein activation differs in these splice variants, as both mGluR1a and 1b can produce qualitatively similar calcium current and M-current inhibition in SCG neurons [11]. Effects of CHPG on both variants was tested primarily to confirm the rather surprising result of CHPG agonism on mGluR1, a result which has not been previously reported despite fairly widespread use of this drug for over a decade [12,26-29]. Indeed, the mGluR1a data shown in Figure 3B is combined data from SCG neurons expressing mGluR1a from two separate, but similar, plasmids. One is an untagged rat mGluR1a, and the other is an N-terminally myc-tagged mGluR1a, both in pCDNA3.1. The data were combined because CHPG acted identically on cells expressing both constructs (not shown). Both constructs (as well as mGluR1b, in pRK5) were tested for responses to CHPG, and sequence-verified. Finally, it should be noted that the results with the mGluR1 constructs were generated using CHPG from two separate sources (Tocris and Ascent, see Materials and Methods) with indistinguishable results (not shown).

## Conclusions

The data presented here indicate that contrary to current dogma, the nominally selective mGluR5 agonist CHPG can act as an agonist for mGluR1 and 5 with similar efficacy and potency, although under some circumstances CHPG may be a poorer activator than Glu of G_q/11_ via mGluR1 compared to mGluR5.

## Abbreviations

CHPG, 2-Chloro-5-hydroxyphenylglycine; mGluR, Metabotropic glutamate receptor; SCG, Superior cervical ganglion; Glu, L-Glutamate; MPEP, 2-Methyl-6-(phenylethynyl)pyridine hydrochloride.

## Competing interests

The author declares that he has no competing interests.

## Authors contributions

All experiments, analysis, writing and editing of the manuscript were performed by PJK.
